# Retraction Note to: Circular RNA circ_0003204 inhibits proliferation, migration and tube formation of endothelial cell in atherosclerosis via miR-370-3p/TGFβR2/phosph-SMAD3 axis

**DOI:** 10.1186/s12929-021-00761-3

**Published:** 2021-10-12

**Authors:** Shanchao Zhang, Guixiang Song, Jing Yuan, Shan Qiao, Shan Xu, Zhihua Si, Yang Yang, Xuxu Xu, Aihua Wang

**Affiliations:** Department of Neurology, The First Affiliated Hospital of Shandong, First Medical University, No. 16766 Jing Shi Road, Jinan, 250014 Shandong China

## Retraction to: J Biomed Sci (2020) 27:11 10.1186/s12929-019-0595-9

The Editor-in-Chief has retracted this article. Concerns were raised regarding a number of figures, specifically:

- Figure 2E: the CTL/NC panel appears to partially overlap with the oxLDL/SIS3 panel of Fig. 5G;
- Figure 2F: the 0 h si-circ_0003204 panel appears to be identical with the 0 h panel of Fig. 5C;
- Figure 2G: the CTL and ox-LLD panels for si-circ_0003204 appear to partially overlap;
- Figure 2G: the ox-LDL panels for si-NC and NC appear to partially overlap;
- Figure 5B: the inhibitor NC and miR-370 inhibitor panels for siRNA NC appear to be identical;
- Figure 5C: the last panel appears to be identical to the second panel of Fig. 5H.

The Editor-in-Chief therefore no longer has confidence in the reliability of the data reported in the article.

Shanchao Zhang, Guixiang Song, Shan Qiao, Zhihua Si, Yang Yang, and Aihua Wang agree to this retraction. Jing Yan, Shan Xu, and Xuxu Xu have not responded to correspondence regarding this retraction.

